# Improving vibration perception in a patient with type 2 diabetes and sensory peripheral neuropathy

**DOI:** 10.4102/sajp.v75i1.602

**Published:** 2019-07-25

**Authors:** Liezel Ennion, Juha Hijmans

**Affiliations:** 1 Department of Physiotherapy, University of the Western Cape, Cape Town, South Africa; 2 University of Groningen, University Medical Center Groningen, Department of Rehabilitation Medicine, Groningen, the Netherlands

**Keywords:** Diabetes type 2, foot ulcers, intervention, vibrating insoles, Stochastic Resonance

## Abstract

**Introduction:**

Diabetes mellitus (DM) and its related sensory peripheral neuropathy (SPN) are the biggest risk factors for foot ulcer formation and lower limb amputation. Reduced vibration perception results in less sensitivity to pressure and is a known risk factor for diabetic foot ulcers. Improving vibration perception in the feet of people with SPN could be protective against foot ulcers. The aim of this study was to determine if a therapeutic vibrating insole, used for 35 consecutive days, could improve vibration perception in a patient with type 2 DM.

**Patient presentation:**

The patient was a 63 year-old male with a medical history of peripheral vascular disease, controlled hypertension, hyperlipidaemia, artherosclerosis and SPN secondary to controlled type 2 diabetes.

**Management and outcome:**

The patient used the insoles for 20 min a day for 35 consecutive days. Vibration perception thresholds were measured four times in total: once at baseline, immediately post intervention, 1 month and 10 months later. Vibration perception threshold decreased with an average of 22 V (range 6 V–34 V) dependant on the tested location and time after intervention. The improvement remained after 1 and 10 months.

**Conclusion:**

The use of a vibrating insole as a therapeutic device improved this patient’s perception of vibration in his feet. Clinically, vibrating insoles potentially might reduce the risk for ulcer formation and subsequent lower limb amputation in patients with DM and SPN.

**Clinical implications:**

Using a vibrating insole therapeutically, can potentially improve the perception of vibration and pressure in patients with sensory peripheral neuropathy secondary to type 2 diabetes mellitus. Improved vibration perception might reduce the risk for diabetic ulcer formation and subsequent lower limb amputation.

## Introduction

Sensory peripheral neuropathy (SPN) is a common complication of diabetes mellitus (DM) (Kärvestedt et al. 2011). The loss of vibration sensation is a major risk factor for developing foot ulcers (Ko & Cha 2012). Foot ulcer, in turn, is the biggest risk factor for lower limb amputation in patients with DM (Lipsky et al. 2011).

Repetitive sensory stimulation (RSS) has been shown to improve sensory perception in patients following stroke, and children with autism, but has not been studied in type 2 diabetic patients with SPN. The brain is known to adapt in response to certain deficits or sensori-motor inputs (Makin et al. 2013). Repeated exposure to stimuli results in changes in the awareness of the sensory system, and its ability to identify certain inputs (Sathian et al. 2013). These changes in acuity are commonly referred to as perceptual learning. The potential application of perceptual learning and adaptations in the brain for rehabilitation is vast.

The same mechanoreceptor in the skin, the Pacinian corpuscle which is responsible for detecting pressure, is also responsible for detecting vibration (as it is in essence pressure applied to the skin). If an intervention aimed at improving a patient with SPN’s ability to perceive vibration is successful, it could be used therapeutically to potentially prevent ulcer formation. At an impairment level, diabetic foot ulcers cause pain, which negatively affects patients’ mobility and balance. Decreased mobility may also worsen the already impaired circulation and prolong ulcer healing time in the diabetic population, increasing the risk of wound infection and lower limb amputation. Challenges with mobility also limit the activities of daily living (social and economic) in which the person can participate. In turn a loss of independence in mobility can lead to psychological problems such as anxiety and depression, decreased participation in society and ultimately social exclusion. Preventing the formation of diabetic foot ulcers may prevent disability across the whole spectrum of impairment, activity and participation and may lead to improved quality of life for persons with DM.

## Ethical considerations

Ethical clearance was obtained from the University of the Western Cape’s Biomedical research ethics committee (BM 16_3_23). Written informed consent was obtained from the participant prior to data collection.

## Patient presentation

A 63-year-old retired male with a medical history of peripheral vascular disease, controlled hypertension, hyperlipidaemia, atherosclerosis and controlled type 2 diabetes mellitus participated in this single-case study. He was diagnosed with diabetes mellitus and peripheral vascular disease 15 years ago. He suffered a transient ischaemic attack at the time, and his carotid disease was discovered. It resulted in a left-sided carotid endarterectomy. The participant had artherosclerosis of both the right and left external iliac arteries, with approximately 90% occlusion, confirmed via angiogram (right more occluded than left). Chronic medication included Diaphage 1 g twice a day, Ciplazar 50 mg, Lomanor 5 mg, Simvastatin 4 mg, Disprin and Dopaquel 200 mg twice a day. The participant had been a smoker for the past 50 years and currently smoked 10 to 15 pipes of tobacco per day. He did not consume any alcohol and had been diagnosed with sensory peripheral neuropathy in both feet secondary to type 2 DM 3 years prior to this study.

The presence of SPN was confirmed with a Bio-Thesiometer (Model PVD-LP). The Bio-Thesiometer is calibrated and considered as the gold standard for diagnosing SPN. The Bio-Thesiometer is considered as the gold standard to detect SPN because of its strong predictive value for diabetic foot ulceration. A vibratory perception threshold (VPT) of 15 V and lower is considered normal or low risk (2.9%) for foot ulceration, while a VPT *of* > 25 V has an increased risk of 19.8% for diabetic ulcers (Young et al. 1994).

## Management

A vibrating insole prototype that delivers a mechanical noise signal at a varied frequency was used as the RSS intervention in this case study. This prototype was similar to the initially developed prototype for a study to enhance balance in patients with peripheral neuropathy. The technical specifications of the insole are described in Hijmans et al. (2007). The participant was requested to wear the insole daily, inside a comfortable shoe in a sitting position. The participant kept a daily log of the time and wore the insole for an average of 20 minutes per day for 35 consecutive days. A log was kept to document the duration of daily wear, in case this varied from the given instructions. The participant continued with his normal activities of daily living in every other aspect. He did not exercise and was largely sedentary due to claudication pain in his legs when he walked more than 100 m at a time. He had been following a low-carbohydrate (Banting) diet for the previous 4 years. No other interventions or medication (apart from his chronic medications listed above) was administered during this period.

A Bio-Thesiometer (Model PVD-LP) was used to establish the participant’s VPT score in volts at baseline. Measures were taken from seven points on the foot. Normal values for each reading are provided in the manual for the Bio-Thesiometer (Table 1). Measurements were repeated at 35 days and 10 months by both the first author and a research assistant after the intervention was initiated to account for any bias.

**TABLE 1:** Results and timeline for prone and supine positions and one month washout.

Timeline	Foot	Left							Right						
	Position	Prone			Supine				Prone			Supine			
	Date	29/1/17	5/3/17	2/4/17	29/1/17	5/3/17	5/3/17	2/4/17	29/1/17	5/3/17	2/4/17	29/1/17	5/3/17	5/3/17	2/4/17
Average of 3× measurements by area of the foot	‘Normal’ value in Volts (V)	Pre-test (V)	Post-test† (V)	Reten-tion (V)	Pre-test (V)	Post-test† (V)	Post-test‡ (V)	Retention (V)	Pre-test (V)	Post-test† (V)	Retention (V)	Pre-test (V)	Post-test† (V)	Post-test‡ (V)	Retention (V)
Base of big toe	8.0	26.0	13.0	11.0	33.0	13.0	7.0	13.0	25.0	13.0	8.0	36.0	11.0	7.0	19.0
Tip of big toe	10.0	38.0	21.0	8.0	40.0	11.0	6.5	11.0	35.0	9.0	8.0	41.0	12.0	5.0	7.0
2nd toe	11.0	18.0	12.0	13.0	17.0	10.0	6.0	7.0	29.0	9.0	13.0	40.0	13.0	6.0	15.0
3rd toe	1.0	28.0	13.0	26.0	32.0	14.0	7.0	19.0	31.0	9.0	22.0	30.0	16.0	6.5	19.0
4th toe	11.0	25.0	10.0	17.0	29.0	14.0	8.0	17.0	39.0	10.0	21.0	35.0	12.0	7.0	15.0
5th toe	10.0	26.0	14.0	29.0	38.0	15.0	7.0	25.0	24.0	13.0	30.0	36.0	15.0	6.0	20.0
Medial instep	8.0	42.0	19.0	30.0	34.0	19.0	7.0	22.0	49.0	15.0	24.0	42.0	13.0	6.0	32.0
Total score over 7 areas	59.0	203.0	102.0	134.0	223.0	96.0	48.5	114.0	232.0	78.0	126.0	260.0	92.0	43.5	127.0
Mean score over 7 areas	8.4	29.0	14.6	19.1	31.9	13.7	6.9	16.3	33.1	11.1	18.0	37.1	13.1	6.2	18.1

† , measurements taken by first author.

‡ , measurements taken by research assistant.

The baseline measurement of the participant’s VPT with the Bio-Thesiometer indicated a mean score of 31 V (unit of measurement for the Bio-Thesiometer) across seven points of both feet in the prone position and a 34.5 V in the supine position. The head of the Bio-Thesiometer was firmly applied to the point of contact on the patient’s foot, and the patient was asked to close his eyes while the first author slowly increased the amplitude of the vibration. The patient then told the first author or research assistant as soon as he detected the vibration, and the first author or research assistant then documented the corresponding reading in volts from the Bio-Thesiometer. Measurements at each point were repeated three times to ensure reliability, and the average was calculated.

## Results

A clinically important (19.7 V) reduction in the VPT was determined between pre-intervention, mean 32.8 V (± 7.1), and post-intervention, mean 13.1 V (± 2.8), scores across the seven points (by the first author). The findings are considered clinically important because the patient no longer fits the criteria for SPN (VPT > 25 V) (Peters et al. 2001). For every 1 V increase in VPT, a 5.6% increase in chance of ulceration is reported (Abbott et al. 1998). Conversely, for every 1 V reduction in VPT, the risk for ulceration should be diminished by 5.6%. Using this formula, the patient should thus have almost no risk for foot ulceration. There was an increase of 4.8 V, mean 17.9 V (± 7.9), in the VPT after the washout period of one month, but this change was not clinically important as the patient was still under the threshold of 20 V, which is considered an increased risk for foot ulceration.

**FIGURE 1:**
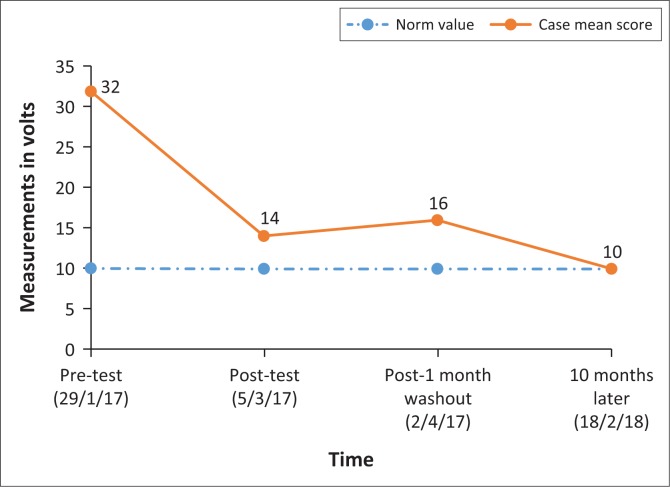
Supine position: change in mean vibratory perception threshold scores (volts) over time.

No clinical differences were detected between the prone and supine positions in the initial tests, or between the left and right foot for any of the measurements; so, values reported are for the left foot as assessed by the first author in the supine position (Figure 1). Subsequent measures were taken only in the supine position as the participant reported being more comfortable in supine. After 10 months (Table 2), the VPT was further reduced by 23.2 V, mean 9.6V (± 3.2), in the supine position. Clinically, the participant reported an overall general improvement in sensation in his feet. He mentioned being able to ‘feel the texture of the carpet’ under his feet which he was not able to feel before the intervention. He also mentioned an overall increased sensitivity in his entire foot and more stability while walking.

**TABLE 2:** Retention of effect over time in supine.

Timeline	Position	Supine											
	Foot	Left						Right					
	Date	29/1/17	5/3/17	5/3/17	2/4/17	18/2/18	18/2/18	29/1	5/3/17	5/3/17	2/4/17	18/2/18	18/2/18
Average of 3× measurements by area of the foot	‘Normal’ value in Volts (V)	Pre-test (V)	Post-test† (V)	Post-test‡ (V)	After washout1 (V)	10 months later† (V)	10 months later† (V)	Pre-test (V)	Post-tes† (V)	Post-test‡ (V)	After washout 1(V)	10 months later† (V)	10 months later‡ (V)
Base of big toe	8.0	33.0	13.0	7.0	13.0	5.0	5.0	36.0	11.0	7.0	19.0	7.0	8.0
Tip of big toe	10.0	40.0	11.0	6.5	11.0	7.0	2.0	41.0	12.0	5.0	7.0	11.0	6.0
2nd toe	11.0	17.0	10.0	6.0	7.0	8.0	5.0	40.0	13.0	6.0	15.0	11.0	6.0
3rd toe	1.0	32.0	14.0	7.0	19.0	10.0	5.0	30.0	16.0	6.5	19.0	8.0	7.0
4th toe	11.0	29.0	14.0	8.0	17.0	7.0	5.0	35.0	12.0	7.0	15.0	9.0	7.0
5th toe	10.0	38.0	15.0	7.0	25.0	12.0	5.0	36.0	15.0	6.0	20.0	8.0	6.0
Medial instep	8.0	34.0	19.0	7.0	22.0	18.0	10.0	42.0	13.0	6.0	32.0	18.0	15.0
Total score over 7 points	70.0	223.0	96.0	48.5	114.0	67.0	37.0	260.0	92.0	43.5	127.0	72.0	55.0
Mean score over 7 points	10.0	31.9	13.7	6.9	16.3	9.6	5.3	37.1	13.1	6.2	18.1	10.3	7.9

† , measurements taken by first author.

‡ , measurements taken by research assistant.

The retention of the effect is new information and has not been investigated or documented before. It seems that in this case, the therapeutic use of vibrating insoles has reversed one of the symptoms of peripheral neuropathy.

## Conclusion

This case study demonstrates a clinical reduction in VPT for a patient with SPN after using a mechanical noise vibrating insole intervention for a period of 20 minutes per day for 35 days. The results from this study may thus have a considerable protecting effect for ulceration.

Owing to the nature of single-case studies, the findings of this study cannot be generalised to the diabetic population but can be used to stimulate further research and discussion on the topic. The physiological mechanism of how this intervention reduced VPT and why the effect is being retained in this case study cannot be explained. It could potentially be as a result of increased sensitivity of the mechanoreceptors on the plantar aspect of the feet and a resultant increase in corticomuscular synchronisation (Trenado et al. 2014). The retention of the effect could point to increased corticomuscular coherence or neuroplastic changes. This effect is, however, currently being investigated with a bigger cohort of patients and in a subsequent functional magnetic resonance imaging study.

The findings of this single-case study are relevant and clinically important as they potentially could result in a reduced risk for ulcer formation in patients with DM and potentially reduce the risk of lower limb amputation. A decreased risk for diabetic foot ulcers can contribute to the decrease of the associated disability by maintaining functional mobility for longer duration. Improved mobility will result in participation in daily and socio-economic activities, and inclusion in society, ultimately resulting in improved quality of life.
